# Extracellular HMGB1 Contributes to the Chronic Cardiac Allograft Vasculopathy/Fibrosis by Modulating TGF-β1 Signaling

**DOI:** 10.3389/fimmu.2021.641973

**Published:** 2021-03-10

**Authors:** Huijuan Zou, Bingxia Ming, Jun Li, Yifan Xiao, Lin Lai, Ming Gao, Yong Xu, Zheng Tan, Feili Gong, Fang Zheng

**Affiliations:** ^1^Department of Immunology, School of Basic Medicine, Tongji Medical College, Huazhong University of Science and Technology, Wuhan, China; ^2^Reproductive Medicine Center, Department of Gynaecology and Obstetrics, The First Affiliated Hospital of Anhui Medical University, Hefei, China; ^3^Department of Rheumatology and Immunology, Tongji Hospital, Tongji Medical College, Huazhong University of Science and Technology, Wuhan, China; ^4^Key Laboratory of Organ Transplantation, Ministry of Education, Chinese Academy of Medical Sciences, Wuhan, China; ^5^NHC Key Laboratory of Organ Transplantation, Chinese Academy of Medical Sciences, Wuhan, China; ^6^Key Laboratory of Organ Transplantation, Chinese Academy of Medical Sciences, Wuhan, China

**Keywords:** HMGB1, TGF-β1, cardiac allograft vasculopathy, transplantation, fibrosis

## Abstract

Cardiac allograft vasculopathy (CAV) charactered with aberrant remodeling and fibrosis usually leads to the loss of graft after heart transplantation. Our previous work has reported that extracellular high-mobility group box 1 (HMGB1) participated in the CAV progression via promoting inflammatory cells infiltration and immune damage. The aim of this study was to investigate the involvement of HMGB1 in the pathogenesis of CAV/fibrosis and potential mechanisms using a chronic cardiac rejection model in mice. We found high levels of transforming growth factor (TGF)-β1 in cardiac allografts after transplantation. Treatment with HMGB1 neutralizing antibody markedly prolonged the allograft survival accompanied by attenuated fibrosis of cardiac allograft, decreased fibroblasts-to-myofibroblasts conversion, and reduced synthesis and release of TGF-β1. In addition, recombinant HMGB1 stimulation promoted release of active TGF-β1 from cardiac fibroblasts and macrophages *in vitro*, and subsequent phosphorylation of Smad2 and Smad3 which were downstream of TGF-β1 signaling. These data indicate that HMGB1 contributes to the CAV/fibrosis via promoting the activation of TGF-β1/Smad signaling. Targeting HMGB1 might become a new therapeutic strategy for inhibiting cardiac allograft fibrosis and dysfunction.

## Introduction

Despite considerable advances have been achieved in controlling acute rejection, the long-term survival of cardiac allograft remains limited by chronic rejection which charactered with cardiac allograft vasculopathy (CAV) (1, 2). The distinctive features of CAV are fibroblasts-to-myofibroblasts conversion and deposition of extracellular matrix (ECM), fibrosis around the blood vessels accompanied by inflammatory cells infiltration, and subsequent lumen constriction resulting in ischemic graft failure (2). Accumulation of lymphocyte cells and macrophages are commonly observed in vascular lesions (3). CAV is the major cause of late allograft dysfunction following heart transplantation, and until now little progress has been made for the prevention of CAV.

Fibrosis is the main event of chronic CAV (4). In fibrotic process, resident cardiac fibroblasts proliferate and develop into matrix-producing α-smooth muscle actin-expressing (α-SMA^+^) myofibroblasts to produce ECM proteins (4, 5). This process is often induced by the factor of transforming growth factor (TGF)-β1. In acute response to injury, TGF-β1 was primarily released from platelets and T cells, this release is important in macrophage and fibroblast chemotaxis to the injured site (6, 7). Functionally activated TGF-β1 produced from an inactive precursor is a crucial mediator of fibrotic disorder, which activates TGF-β1 signaling through a classical SMAD pathway (7) or an alternative SMAD independent pathway (8).

High-mobility group box 1 (HMGB1), a ubiquitous non-histone nuclear protein, functions as a danger signal in extracellular circumstances and participates in various inflammatory diseases, tissue injury and fibrotic diseases (9–14). Accumulating studies have reported extracellular HMGB1 contributing to the acute cardiac allograft rejection (15–19). Our previous work has shown high level of extracellular HMGB1 in chronic cardiac allograft, and that blockade of HMGB1 markedly attenuated the CAV via inhibiting the inflammatory cells infiltration and immune damage (17). In heart transplantation recipients, a strong association between the expression of TGF-β in cardiac biopsy specimens and the development of vasculopathy was observed (20). However, whether HMGB1 could directly affect the fibrosis of CAV is unknown. Therefore, we hypothesize that extracellular HMGB1 might promote CAV/fibrosis via enhancing TGF-β1 signaling in allograft.

Using a single MHC Class II (MHC-II)-mismatched mouse heart transplantation model to reflect CAV, we observed that active TGF-β1 was increased in cardiac allograft. Blockade of HMGB1 prolonged the allograft survival and CAV/fibrosis via inhibiting the synthesis and release of TGF-β1, and subsequent activation of TGF-β1/Smad signaling. Therefore, targeting HMGB1 might not only prevent inflammatory damage of allografts, but also become a new therapeutic strategy for inhibiting cardiac allograft fibrosis and dysfunction.

## Materials and Methods

### Mice

Female C57BL/6 (B6, H-2^b^) mice, aged 6–8 weeks, were obtained from Hubei Research Center of Laboratory Animals (Wuhan, China). B6.C-H-2^bm12^KhEg (bm12, H-2^bm12^) females, strained of mice B6 arose through a spontaneous mutation in the MHC class II molecule, I-A^b^, were purchased from the Jackson Laboratory (Bar Harbor, ME, USA). All experimental mice were housed in humidity and temperature controlled specific pathogen-free conditions in the animal facility with autoclaved sterile diet and water. All experiments were performed in compliance with the guidelines of Institutional Animal Care and Use Committee (IACUC) at Tongji Medical College (Wuhan, China).

### Cardiac Transplantation

Heterotopic cardiac transplantation was performed using a microsurgical technique as previously described by Corry et al. (21, 22). In single MHC class-II mismatched heart transplantation models, bm12 mice were used as donors and B6 mice were used as recipients. Briefly, the cardiac allograft (from bm12 mice) was transplanted in the abdominal cavity by anastomosing the aorta and pulmonary artery of the graft end-to-side to the recipient's (B6 mice) aorta and vena cava, respectively. This is an established murine model of CAV. After surgery, the strength and quality of allograft impulses were assessed by daily abdominal palpation. CAV was defined as the change of detectable heart beating and verified by histological examination.

### HMGB1 Antibody Treatment

Anti-HMGB1 neutralizing monoclonal antibody (isotype mouse IgG) (HMGB1 mAb) was obtained by the Institute of Biophysics, Chinese Academy of Science (Beijing, China) (17). Briefly, two-hundred microgram of HMGB1 mAb was intraperitoneally (i.p.) injected into recipients from the day before transplantation, then twice a week till week 4 after transplantation. Recipients received the same amount of normal mouse IgG (Sigma-Aldrich, Saint Louis, MO, USA) were served as controls.

### Western Blotting

Total proteins were extracted from cardiac grafts or cell lysates after recombinant HMGB1 (rHMGB1) (Sigma-Aldrich, Saint Louis, MO, USA) stimulation, and subjected to immunoblots as well as incubation with mouse monoclonal anti-TGF-β1 (1:500, Abcam plc, Cambridge, UK), rabbit monoclonal anti-α-SMA (1:10,000, Abcam plc, Cambridge, UK), rabbit polyclonal anti-p-Smad2 (1:200, Santa Cruz Biotechnology, Inc., Dallas, USA), rabbit polyclonal anti-p-Smad3 (1:500, Sangon Biotechnology Co., Ltd., Shanghai, China), rabbit polyclonal anti-Smad2 (1:500, Shanghai Sangon Biotechnology Co., Ltd., Shanghai, China), rabbit polyclonal anti-Smad3 (1:500, Shanghai Sangon Biotechnology Co., Ltd., Shanghai, China) or rabbit polyclonal anti-GAPDH (1:1,000, ZSGQ-BIO, Beijing Zhong Shan Jin Qiao Biotechnology Co., Ltd., Beijing, China). Blots were visualized by an ECL system (Pierce Biotechnology, Rockford, USA) after incubation with horseradish peroxidase (HRP)-conjugated secondary antibodies (1:5,000, Santa Cruz Biotechnology, Inc., Dallas, USA), and were quantified by densitometry using an image analysis program (IMAGE J, NIH, Bethesda, USA).

### Immunohistochemistry

5-μm-thick paraffin sections of grafts harvested at week 8 were incubated with mouse monoclonal anti-TGF-β1 (1:250, Abcam plc, Cambridge, UK) overnight at 4°C, then stained using the streptavidin/peroxidase histostain^TM^-plus kit (Beijing Zhong Shan Jin Qiao Biotechnology Co., Ltd., Beijing, China) according to the manufacturer's recommendations. The slides were evaluated using Zeiss (Carl Zeiss AG, Oberkochen, Germany) microscope.

### Graft Histological Analysis

Grafts harvested at pointed time were fixed in 4% paraformaldehyde, embedded in paraffin and cut into 5-μm-thick sections for Masson staining to evaluate collagen expression. Masson staining was performed according to the Masson-Goldner trichrome kit (Servicebio Biotechnology, Wuhan, China).

### ELISA

Blood samples, allograft homogenates and cell culture supernatants were collected at indicated time. The concentration of free active and total TGF-β1 in the sera, heart homogenates or cell culture supernatants were determined by ELISA kits (Biolegend, SanDiego, CA, USA) according to the manufacturer's instructions.

### Immunofluorescence

Subcellular localization of TGF-β1 and F4/80 or α-SMA proteins in allografts was identified by using immunofluorescent staining. Formalin-fixed, paraffin embedded tissue sections washed with PBS, and blocked with blocking reagent for 2 h. Then the sections were incubated overnight at 4°C with primary antibodies against either TGF-β1 (1:250, Abcam plc, Cambridge, UK), F4/80 (1:400, Biolegend, San Diego, CA, USA) or α-SMA (1:250, Abcam plc, Cambridge, UK). Subsequently, the bound signal was visualized by Alexa Fluor 488- anti-rabbit antibody or 594-conjugated (Molecular Probe, Eugene, OR). Nuclei were counterstained with DAPI (0.1 μg/ml, Sigma-Aldrich, St Louis, MO, USA). The co-localization of these indicators were observed by confocal microscopy (Carl Zeiss AG, Oberkochen, Germany).

### Isolation of Fibroblasts From Neonatal Mouse Heart

Hearts from neonatal mice were pooled and treated as an individual sample. Hearts were divided into atrium and ventricle under the microscope (Stereomaster, Fisher Scientific, Waltham, MA) then dissociated into mononuclear cells as previously described with minor modification. Briefly, hearts were minced with dissecting scissors into ≤3 mm pieces and digested with 2 mg/mL Collagenase type IV (Worthington, Lakewood, NJ) and 0.1 mg/ml DNase I (Sigma-Aldrich, Saint Louis, MO, USA) in PBS at 37°C for 45 min with agitation every 15 min. After 45 min, enzymes were neutralized by adding twice the original volume of Ham's F10 with L-Glutamine (HyClone, Logan, UT, USA) and 15% horse serum (HyClone, Logan, UT, USA), filtered through sterile 70 mnylonmeshcell strainer (ThermoFisher Scientifics, Waltham, MA, USA), centrifuged at 300 g for 5 min. Single cell suspension was resuspended in PBS, counted and transferred to culture immediately.

### Generation of Mouse BMDMs

Bone marrow-derived macrophages (BMDMs) were propagated from mouse bone marrow as described previously (23). After 7 days of culture, the cells were stimulated with mouse rHMGB1 for 48 h or 0, 10, 30, 60 min. The supernatants were collected for cytokines analysis by ELISA and the cells were harvested for western blotting.

### Statistical Analysis

Allograft survival curve was generated by the Log-rank and Gehan-Breslow-Wilcoxon test. Other data are presented as mean ± standard error of the mean (SEM), and comparison between two groups was performed using a two-tailed Student's *t* test. The difference among groups was conducted by one-way analysis of variance (ANOVA) followed by Bonferroni correction. Values of *p* < 0.05 were considered statistically significant.

## Results

### Blockade of HMGB1 Prolonged Chronic Cardiac Allograft Survival and Attenuated Allograft Fibrosis

**Figure 1:**
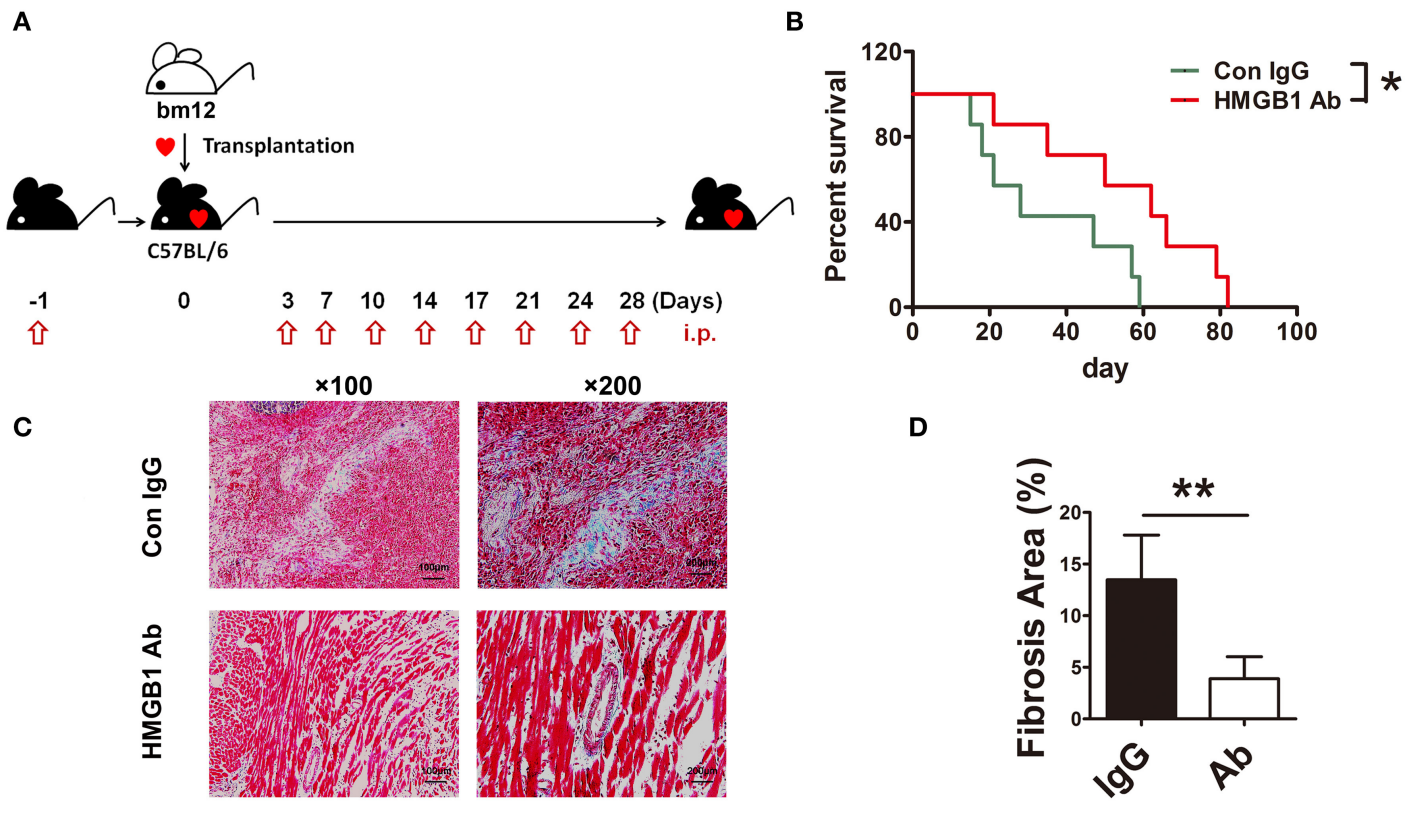
The effects of HMGB1 mAb on murine cardiac allograft survival and fibrosis. HMGB1 mAb or control mouse IgG (200 μg each time) was intraperitoneally (i.p.) injected into the allogeneic recipients, from the day before transplantation and then twice a week untill week 4 after transplantation. Allograft impulse was assessed by daily abdominal palpation and cardiac tissue samples were collected at indicated time after transplantation. **(A)** Therapeutic strategy about HMGB1 mAb. **(B)** The allograft survival time were evaluated in two groups (n=7/group). *: *p*<0.05, vs. the IgG group. **(C)** Fibrotic features of allografts from each experimental group were evaluated at week 8 post-transplant by Masson staining. Scale bar: 100 μm and 200 μm. **(D)** Quantitative analysis of cardiac fibrosis area by Masson staining. Values were shown as mean ± SEM (n=3/group). **: *p*<0.01. All data were representative of two or three independent experiments.

To investigate the role of HMGB1 in the fibrosis of cardiac allograft, the heart of bm12 mouse was transplanted to the B6 mouse, which was called MHC-II mismatched model charactered by chronic allograft fibrosis. The mouse HMGB1 mAb, a specific blockade for HMGB1, was administered to recipients as schematized in Figure 1A. The same amount of IgG administered to the recipients was served as control. Allograft survival was monitored daily. The survival time of allografts in HMGB1 mAb treated recipients was significantly longer than that in IgG isotype treated recipients (Figure 1B). Allografts especially the affected vascular site from HMGB1 mAb treated recipients showed a marked decrease in the expression of collagenous fibers (which stained blue) at week 8 post-transplant (Figures 1C,D). These data indicate that HMGB1 was involved in the vasculopathy/fibrosis of cardiac allografts.

### TGF-β1 Was Increased in Allograft After Cardiac Transplantation

**Figure 2:**
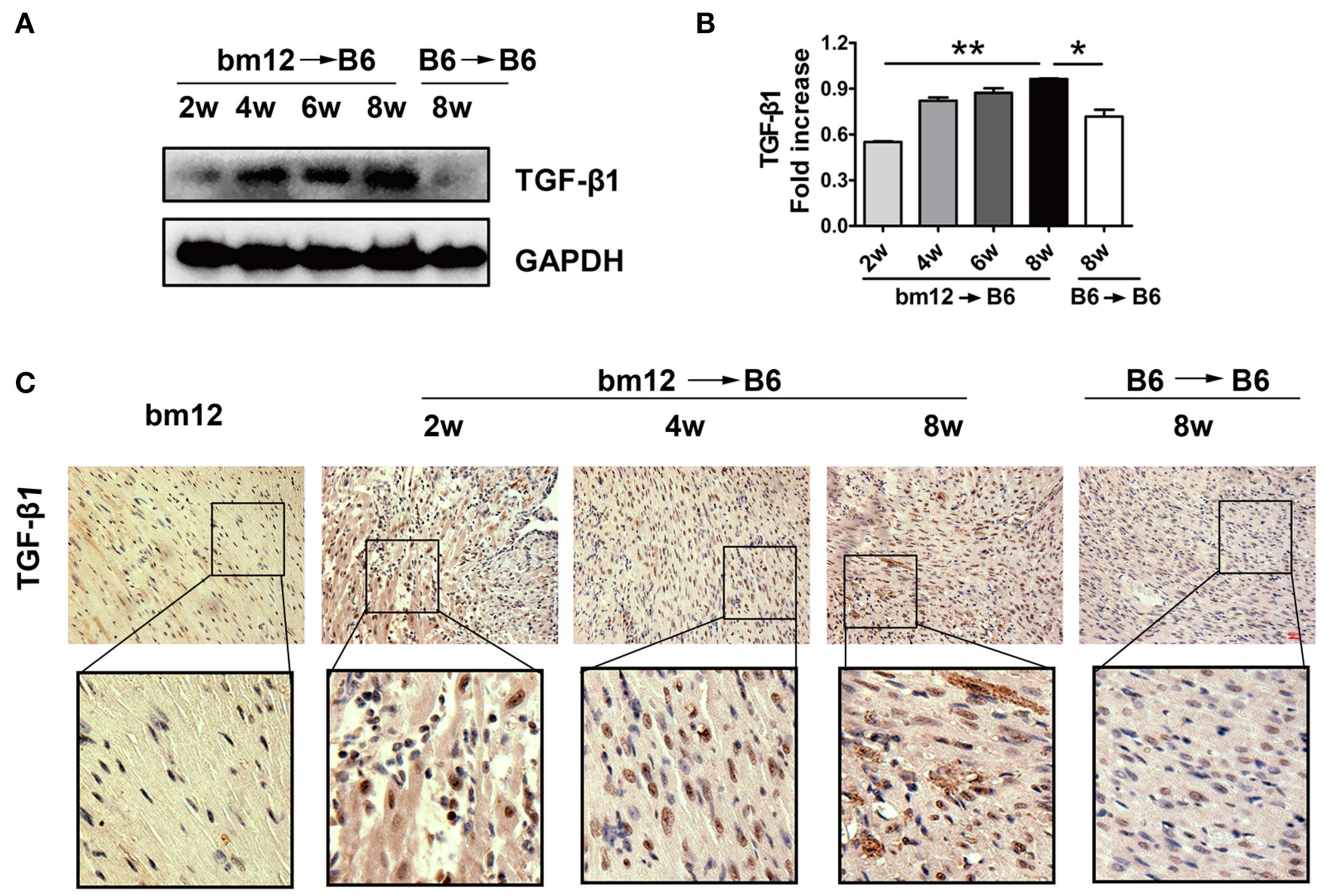
The expression of TGF-β1 in cardiac allografts. Cardiac tissue samples were collected at indicated time after transplantation. **(A)** Expression of TGF-β1 protein in allografts. **(B)** The relative quantity of TGF-β1 protein normalized to GAPDH. The data of **(B)** was shown as mean ± SEM (n=3/group). *: *p*<0.05; **: *p*<0.01, ***: *p*<0.001. **(C)** The localization of TGF-β1 in allografts was examined by immunohistochemistry staining. TGF-β1 was stained brown and the nuclei was counterstained blue. Upper image: scale bar: 50 μm; Lower image: enlarged view of the box above. The data were representative of three independent experiments.

TGF-β1 has been reported to modulate the pathological process of fibrotic diseases. To verify the expression of TGF-β1 during CAV progression, we examined TGF-β1 level in allografts after heart transplantation. The protein level of TGF-β1 in allografts continuously increased in the development of CAV (Figures 2A,B). Meanwhile, compared with syngeneic graft, HMGB1 positive cells in the allografts markedly elevated at week 2, 4, 8 post transplantation (Figure 2C). The results suggest that TGF-β1 was associated with the pathogenesis of HMGB1-related CAV/fibrosis.

### Blockade of HMGB1 Attenuated the Synthesis and Release of TGF-β1 in Graft

**Figure 3:**
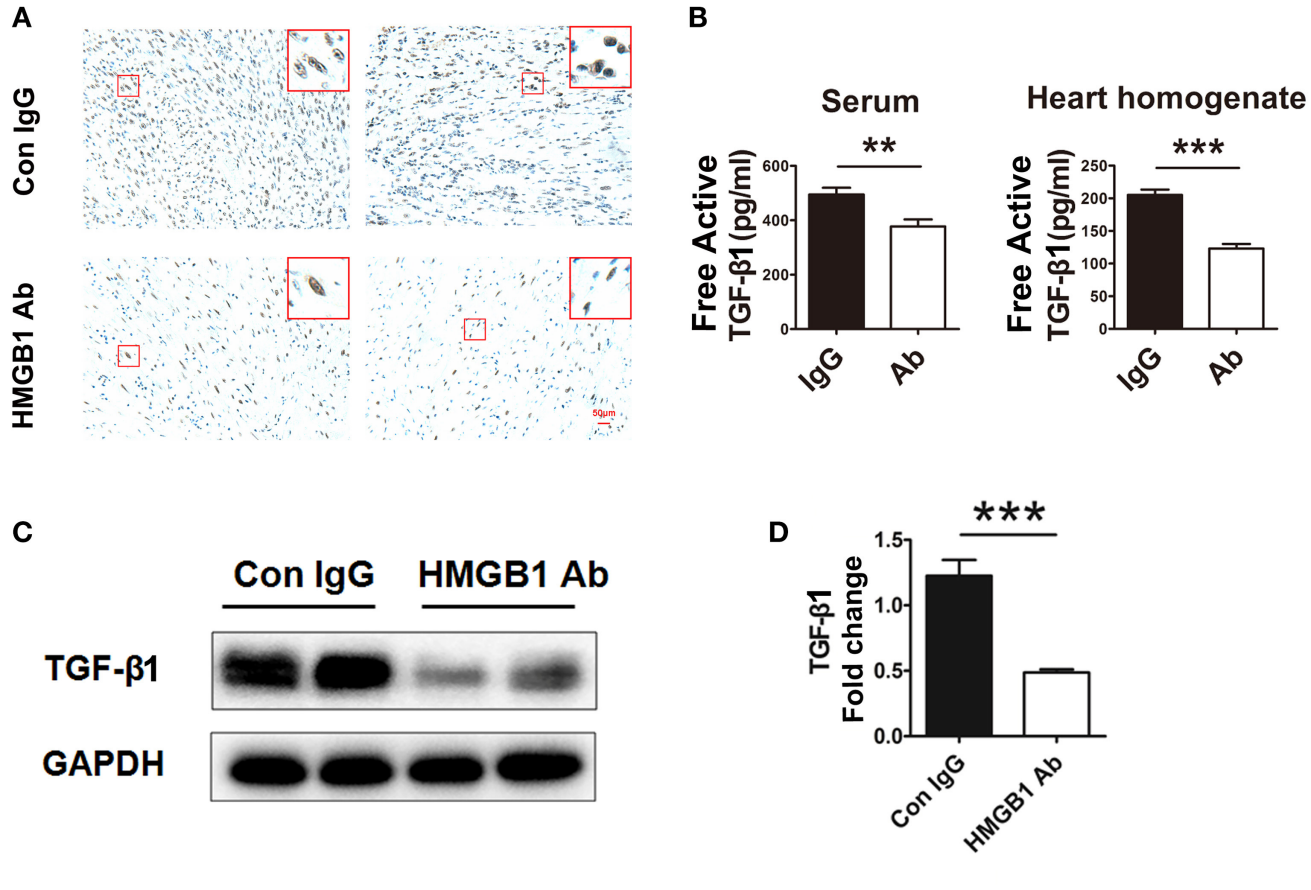
HMGB1 blockade attenuated the synthesis and release of TGF-β1 in allografts. **(A)** Representative images showed the protein expression of TGF-β1 (brown arrows) by immunohistochemical analysis in cardiac allografts at week 8 post transplantation in two groups. Scale bar: 50 μm. **(B)** Concentration of TGF-β1 in recipients' sera and the allograft homogenate at week 8 post-transplant tested by ELISA. Values were expressed as means ± SEM (*n* = 4–8/group). **: *p*<0.01; ***: *p*<0.001, vs. the IgG group. **(C)** The protein expression of TGF-β1 by western blotting after HMGB1 mAb administration. **(D)** The relative quantity of TGF-β1 protein normalized to GAPDH. Data were shown as mean ± SEM. ***: *p*<0.001, vs. the IgG group. All data were representative of three independent experiments.

It has been reported that HMGB1 was involved in the activation of TGF-β1/Smad signaling in abnormal lung remodeling. To investigate the effect of HMGB1 on TGF-β1 production in fibrotic cardiac allografts, the change of TGF-β1 after HMGB1 mAb treatment was examined. As illustrated in Figure 3A, the number of HMGB1 positive cells in HMGB1 mAb treated allografts was markedly decreased compared with that in IgG control group. The amount of free active TGF-β1 in serum of receipt mice and allograft homogenate (Figure 3B), and total TGF-β1 in allografts (Figures 3C,D), were reduced after HMGB1 mAb treatment. These data indicate that HMGB1 affected the production of TGF-β1 and that the pathogenesis of HMGB1 in the fibrosis of cardiac allograft might partly depend on TGF-β1.

### HMGB1 Promoted the Release of TGF-β1 From Cardiac Fibroblasts

**Figure 4:**
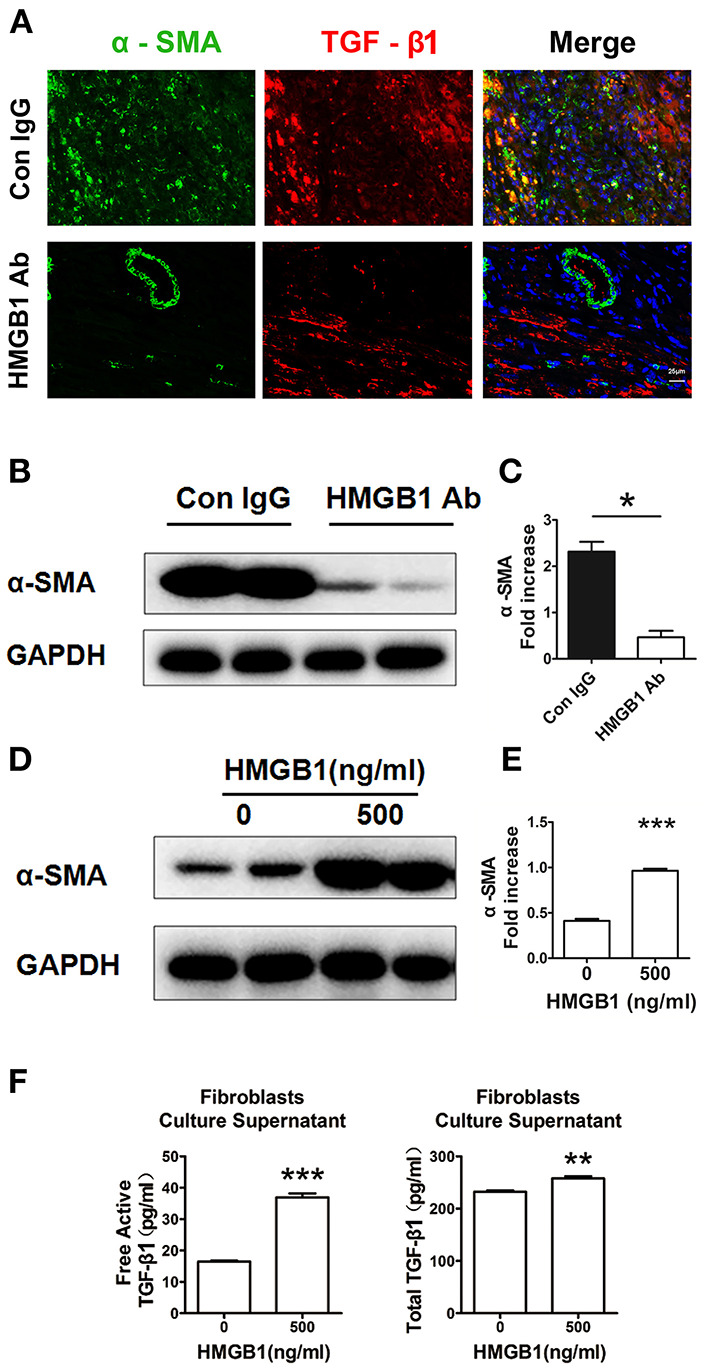
Extracellular HMGB1 promoted the release of TGF-β1 from fibroblasts. **(A)** The expression of TGF-β1 on myofibroblasts (α-SMA+) in the allograft at week 8 posttransplant and HMGB1 mAb treatment. α-SMA was labeled with green fluorescence, TGF-β1 was labeled with red color and nuclei were stained with DAPI in blue. Scale bar: 25 μm. **(B)** The protein expression of α-SMA by western blotting after HMGB1 mAb administration and **(C)** the relative quantity of α-SMA protein normalized to GAPDH in grafts. After 16 h of serum starvation, stimulation with rHMGB1 for 48 h at dose of 0 or 500 ng/ml in media containing 0.5% FBS, **(D)** western blotting analysis of α-SMA protein expression in cultured cardiac fibroblasts, **(E)** the relative quantity of α-SMA protein normalized to GAPDH, **(F)** free active and total TGF-β1 in the supernatants of fibroblasts after rHMGB1 stimulation detected by ELISA. Data were expressed as mean ± SEM (n=3/group) and representative of at least three separate experiments. *: *p*<0.05; **: *p*<0.01; ***: *p*<0.001, vs. negative control.

Myofibroblasts transferred from fibroblasts are the major source of extracellular matrix (ECM) and TGF-β1 in the fibrosis progression, we then detected the direct effect of HMGB1 on fibroblasts. As shown in Figure 4A, myofibroblasts (α-SMA^+^) expressed TGF-β1 in the allograft at week 8 post transplantation. After HMGB1 mAb treatment, the expression of TGF-β1 in myofibroblasts was reduced. Myofibroblast marker α-SMA was significantly reduced in allograft after HMGB1 mAb administration (Figures 4B,C). *In vitro*, exogenous HMGB1 stimulation for 48 h promoted the expression of α-SMA in cultured neonatal cardiac fibroblasts (Figures 4D,E) and elevated the levels of free active and total TGF-β1 in culture supernatants (Figure 4F). These results indicate that HMGB1 promoted the transform of fibroblasts to myofibroblasts and the release of TGF-β1 from myofibroblasts.

### HMGB1 Enhanced Macrophage Released TGF-β1

**Figure 5:**
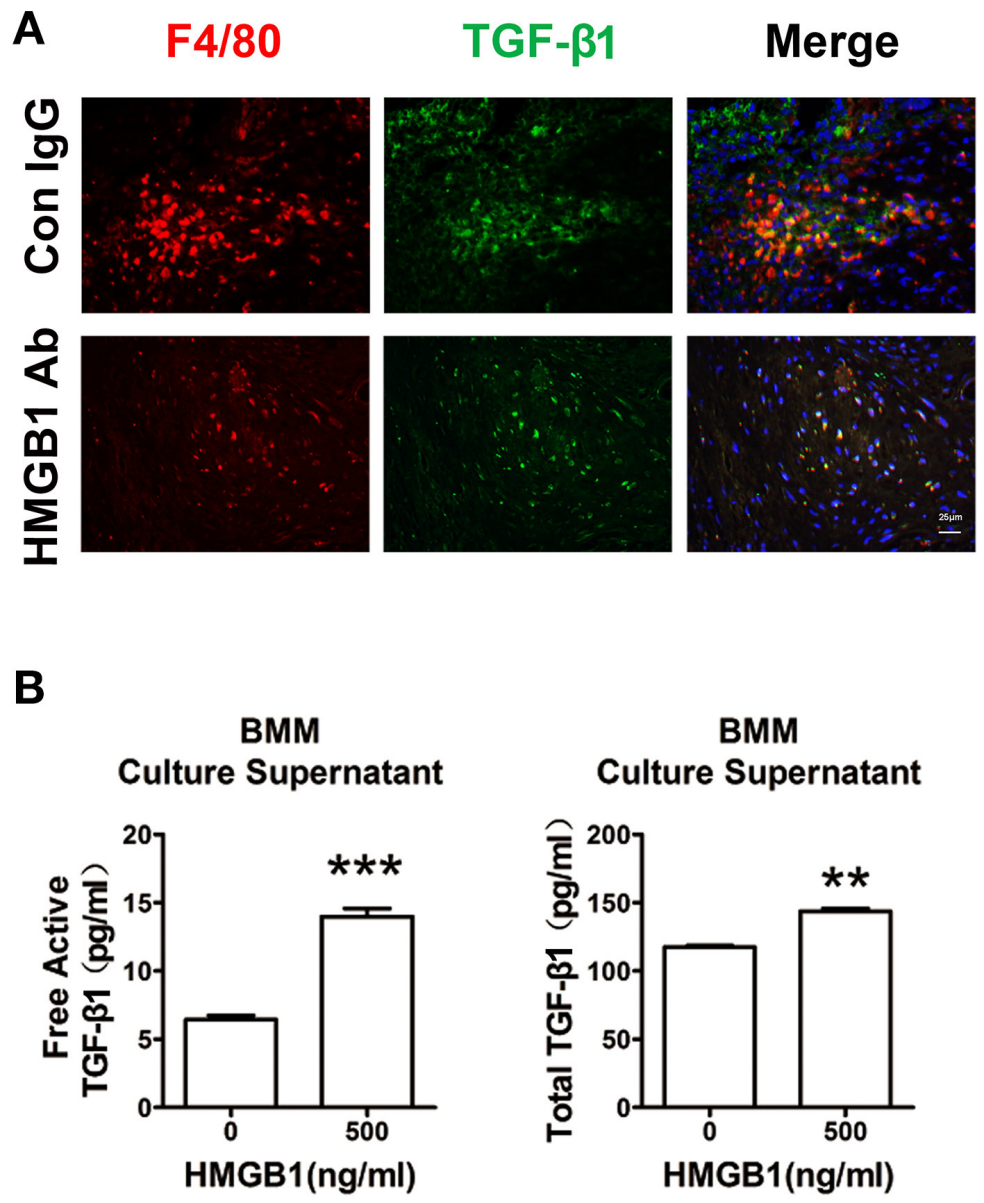
Extracellular HMGB1 enhanced macrophage released TGF-β1. **(A)** The expression of TGF-β1 on macrophages (F4/80+) in the allograft at week 8 posttransplant and HMGB1 mAb treatment. F4/80 was labeled with red color, TGF-β1 was labeled with green fluorescence and nuclei were stained with DAPI in blue. Scale bar: 25 μm. **(B)** After 16 h of serum starvation, BMMs were treated with recombinant HMGB1 at 500 ng/mL for 48h in the presence of 0.5% FBS. Free active and total TGF-β1 in the supernatants of BMMs were detected by ELISA. Data were expressed as means ± SEM (*n* = 3/group). **: *p*<0.01; ***: *p*<0.001, vs. negative control. All data were representative of three independent experiments.

Our previous work has shown that macrophage was the major cell type infiltrated in the cardiac allografts, we then detected its role in the fibrosis of allograft. As shown in Figure 5A, macrophages (F4/80+) expressed TGF-β1 in the allograft at week 8 post heart transplantation. After HMGB1 mAb treatment, the expression of TGF-β1 in macrophages was markedly reduced. *In vitro*, HMGB1 treated bone marrow-derived macrophages (BMMs) released higher levels of free active and total TGF-β1 to the culture supernatant (Figure 5B). These results indicate that HMGB1 promoted the release of TGF-β1 from macrophage.

### HMGB1 Triggered Smad2 and Smad3 Phosphorylation in Macrophages and Cardiac Fibroblasts

**Figure 6:**
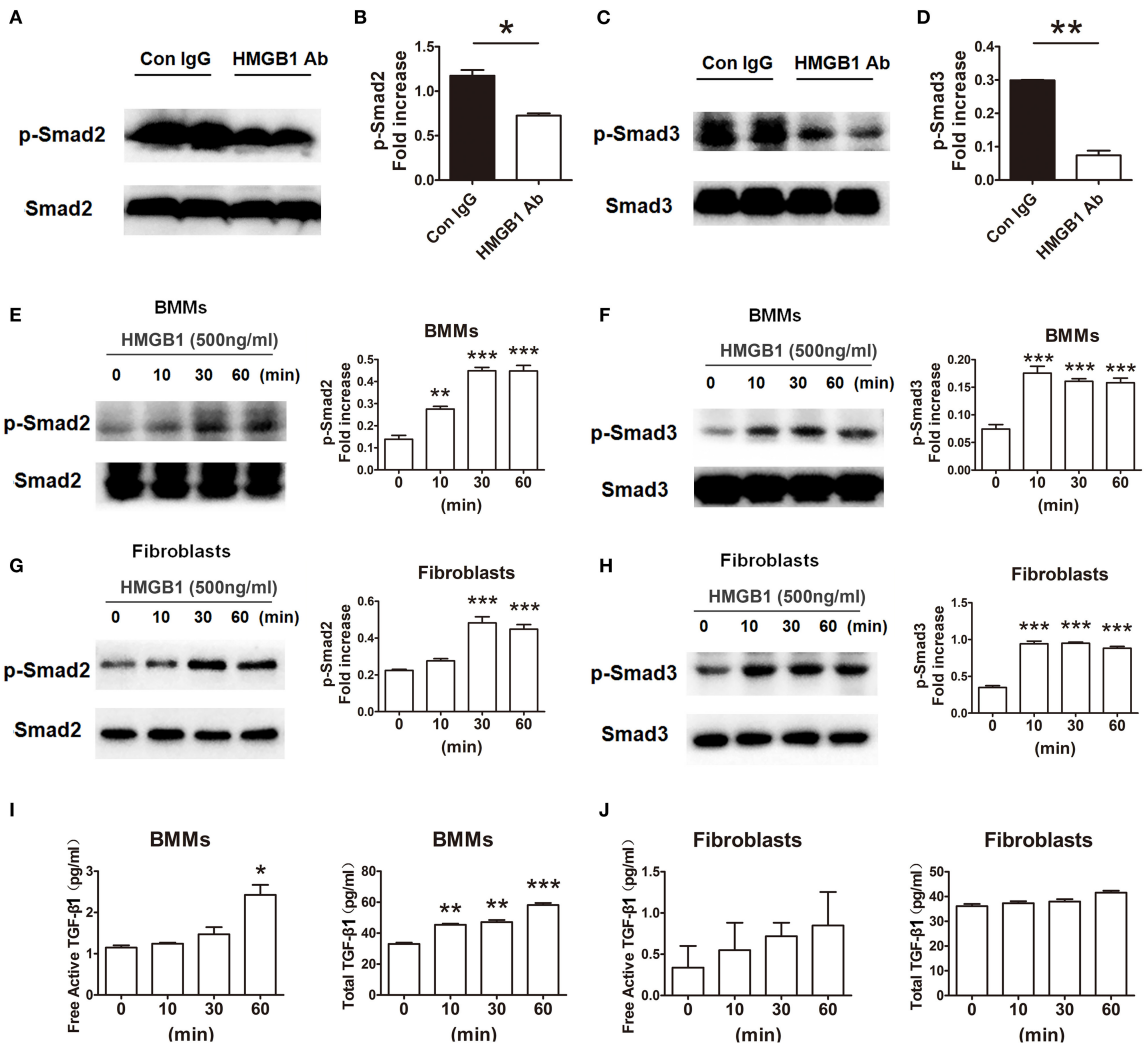
HMGB1 induced phosphorylation of Smad2 and Smad3 in macrophages and fibroblasts. **(A,C)** The protein expression of p-Smad2 and p-Smad3 by western blot after HMGB1 Ab administration. **(B,D)** The relative quantity of p-Smad2, p-Smad3 protein normalized to total Smad2 and Smad3. Data were shown as mean ± SEM and represented of at least three separate experiments. *: *p*<0.05; **: *p*<0.01, ***: *p*<0.001, vs. the IgG group. All data were representative of three independent experiments. **(E–H)** After 16 h of serum starvation, cultured macrophages and fibroblasts were treated with rHMGB1 for 0, 10, 30 and 60 min in the presence of 0.5% FBS. The phosphorylation of Smad2 and Smad3 in BMMs **(E,F)** and fibroblasts **(G,H)** was observed with western blot. The relative quantity of p-Smad2 and p-Smad3 normalized to total Smad2 and Smad3, respectively. The data were shown as mean ± SEM (*n* = 3/group). **: *p*<0.01; ***: *p*<0.001, vs. negative control. All data were representative of three independent experiments. **(I,J)** ELISA to detect for free active and total TGF-β1 levels of BMMs **(I)** and fibroblasts **(J)** cultured supernatant. The data were shown as mean ± SEM (*n* = 3/group). *: *p*<0.05; **: *p*<0.01; ***: *p*<0.001, vs. negative control. All data were representative of two independent experiments.

In order to research the effect of HMGB1 on the downstream signaling of TGF-β1, the activation of TGF-β1/Smad signaling were observed. As illustrated in Figures 6A–D, the expression of p-Smad2 and p-Smad3 in the allografts were significantly decreased in HMGB1 mAb treated group compared with that in IgG control group. In addition, exogenous HMGB1 stimulation obviously induced Smad2 and Smad3 phosphorylation as normalized to total Smad2/Smad3 level in BMMs, even early at 10 min after HMGB1 treatment (Figures 6E,F), and similar results were observed in fibroblasts (Figures 6G,H). Simultaneously, a significant increase of free active TGF-β1 at 60 min and total TGF-β1 at 10 min in the supernatants of BMMs after HMGB1 treatment (Figure 6I), but HMGB1 treatment for 1 h did not change the release of TGF-β1 from fibroblasts (Figure 6J). These data indicate that HMGB1 might induce Smad2/3 phosphorylation in fibroblasts in a TGF-β1 dependent or independent method.

## Discussion

In our previous study and other works, it has been reported the involvement of HMGB1 in the development of cardiac allograft rejection via promoting the inflammatory cells infiltration and immune damage (24, 25). However, whether HMGB1 contributes to the fibrotic damage of cardiac allograft is unclear. Now, we showed a connection between HMGB1 and TGF-β1 in a single MHC-II–mismatched heart transplantation mouse model, and also found extracellular HMGB1 promoting the synthesis and release of TGF-β1 and subsequent TGF-β1/Smad signaling in allograft fibroblasts and macrophages. Blockade of HMGB1 with neutralizing mAb prolonged cardiac allograft survival and alleviated CAV/fibrosis.

The common characteristics of CAV are intimal thickening, medial apoptosis, adventitial deposition of ECM and fibrosis, accumulation of lymphocyte cells and macrophages in affected vascular (3). The adventitia of CAV is presented with fibroblasts-to-myofibroblasts conversion, ECM production and inflammatory cells infiltration (4). The lumen of the affected artery is consisted of an endothelial cell monolayer and a mononuclear cell infiltrate. The latter includes mainly T cells and macrophages, and innate lymphoid cells and other myeloid cell types such as dendritic cells at low frequency (2). In other acute injured condition, released TGF-β1 from platelets plays a critical role in macrophage and fibroblast chemotaxis to the wound site (7). In chronic cardiac allograft rejection, resident fibroblasts are primarily responsible for excessive production of ECM proteins, although other cellular sources such as endothelial cells may also contribute to fibrosis (26, 27). In this study, decreased number of F4/80^+^ macrophage was found in anti-HMGB1-treated than in IgG-treated cardiac grafts (Figure 5). Therefore, we mainly focused on fibroblasts and macrophages to explore the effect of HMGB1 on CAV/fibrosis in allografts. Whether this macrophage infiltration was attributed to monocyte-macrophage chemotaxis or differentiation from resident macrophage needs to be further confirmed. The macrophages in allografts may be M2 phenotype, as it has been reported in other conditions that M2 macrophages participated in fibrosis progression (28, 29).

The TGF-β superfamily is an important mediator of tissue repair and fibrotic disorders, and TGF-β/Smad signaling has been implicated in these processes (30, 31). In diabetic cardiopathy mouse models, TGF-β/Smad signaling mediates the cardiac fibrosis (32, 33). In heart transplantation recipient patients, the expression of TGF-β in cardiac biopsy specimens was strongly associated with the development of vasculopathy (20). A positive association between HMGB1 and TGF-β1 expression was identified in chronic allograft nephropathy (11). TGF-β has 3 isoforms including TGF-β1, -β2, and -β3. Functionally activated TGF-β, produced from an inactive precursor (pro-TGF-β) by convertase, can participate in cellular response via binding to transforming growth factor-β receptor. TGF-β signaling initiates via the downstream SMAD pathway or an alternative SMAD independent pathway. Among the isoforms, TGF-β1 is the most prevalent and thought to be the most biologically relevant (7, 34). Thus, we explored the effect of HMGB1 on TGF-β1 signaling in CAV/fibrosis.

In this study, we found that the expression of TGF-β1 was increased during the CAV progression, and that blockade of HMGB1 significantly downregulated the fibroblasts-to-myofibroblasts conversion and the synthesis and release of TGF-β1 in cardiac allografts. Moreover, extracellular rHMGB1 stimulation promoted the synthesis and release of TGF-β1 in fibroblasts and macrophages. Interestingly, we observed that exogenous HMGB1 stimulation for 48 h induced the release of active TGF-β1 (Figure 4F), as it has been shown in lung fibroblasts (35), while the stimulation of HMGB1 for 1h directly triggered the Smad2 and Smad3 phosphorylation and unchanged the release of active TGF-β1 in cardiac fibroblasts *in vitro* (Figure 6J). These data suggest that HMGB1 might be involved in the CAV/fibrosis via promoting TGF-β1/Smad signaling by a TGF-β1 dependent or independent method. Further investigation should be performed to support this thesis, it may be associated with the expression of HMGB1 receptor on the cardiac fibroblasts (36, 37).

TGF-β regulates cell growth and differentiation, apoptosis, angiogenesis and ECM production (34). It is also commonly viewed as the major immunosuppressive cytokine that prevents immunity through its anti-inflammatory and antiproliferative properties (38, 39). As a major profibrotic factor in fibrotic disorders, anti-TGF-β treatment prevented skin and lung fibrosis in a mouse model for scleroderma (40); administration of TGF-β1-directed antibody prevented ECM matrix protein expression from injured vascular smooth muscle cell lines (41). Studies also showed that TGF-β1 mAb treatment did not prevent the progression of diabetic nephropathy (42) and anti-TGF-β treatment for renal fibrosis was ineffective and non-specific (43). CAV is associated with overexpression of TGF-β1 (20, 27, 44). Inhibition of TGF-β1 may be effective in preventing the fibrosis but may delete its anti-inflammatory effect in chronic CAV, which needs further study to confirm. Based on our previous work and present study, it was found that HMGB1 promoted the immune inflammatory damage and chronic CAV/fibrosis, and that blockade of HMGB1 significantly prolonged the cardiac allograft survival. Therefore, inhibition of extracellular HMGB1 might be a promising strategy for the prevention of CAV after heart transplantation.

In conclusion, our work in a chronic cardiac allograft rejection mouse model demonstrated that extracellular HMGB1 was involved in chronic CAV and fibrosis via promoting TGF-β1 signaling after heart transplantation. Blockade of HMGB1 might represent a promising therapeutic target for the simultaneous inhibition of allograft chronic inflammatory damage and vasculopathy/fibrosis progression.

## Data Availability Statement

The raw data supporting the conclusions of this article will be made available by the authors, without undue reservation.

## Ethics Statement

The animal study was reviewed and approved by the Tongji Medical College Animal Care and Use Committee.

## Author Contributions

FZ designed the study. HZ and BM performed the experiments and analyzed the data. JL, YX, LL, and MG helped for bleeding the mice and samples acquired. ZT, YX, and FG contributed to the interpretation of the data. HZ and BM wrote the paper. All authors read and approved the final manuscript.

## Conflict of Interest

The authors declare that the research was conducted in the absence of any commercial or financial relationships that could be construed as a potential conflict of interest.
